# Development of an Innovative and Green Method to Obtain Nanoparticles in Aqueous Solution from Carbon-Based Waste Ashes

**DOI:** 10.3390/nano11030577

**Published:** 2021-02-25

**Authors:** Raffaella Striani, Enrica Stasi, Antonella Giuri, Miriam Seiti, Eleonora Ferraris, Carola Esposito Corcione

**Affiliations:** 1 Dipartimento di Ingegneria dell’Innovazione, Università del Salento, 73100 Lecce, Italy; raffaella.striani@unisalento.it (R.S.); enrica.stasi@unisalento.it (E.S.); 2 Department of Mechanical Engineering, Campus de Nayer, KU Leuven, 2860 Sint-Katelijne-Waver, Belgium; miriam.seiti@kuleuven.be (M.S.); eleonora.ferraris@kuleuven.be (E.F.); 3 Istituto di Nanotecnologia CNR-Nanotec, Polo di Nanotecnologia c/o Campus Ecotekne, Via Monteroni, 73100 Lecce, Italy; antonella.giuri@unisalento.it

**Keywords:** recycling, circular economy, nanometric carbon-based ashes, AJ^®^P

## Abstract

In this study, an original and green procedure to produce water-based solutions containing nanometric recycled carbon particles is proposed. The nanometric particles are obtained starting from carbon waste ashes, produced by the wooden biomass pyro-gasification plant CMD (Costruzioni motori diesel) ECO20. The latter is an integrated system combining a downdraft gasifier, a spark-ignition internal combustion engine, an electric generator and syngas cleaning devices, and it can produce electric and thermal power up to 20 kWe and 40 kWth. The carbon-based ashes (CA) produced by the CMD ECO20 plant were, first, characterized by using differential scanning calorimetry (DSC) and microcomputed tomography (microCT). Afterward, they were reduced in powder by using a milling mortar and analyzed by scanning electron microscopy (SEM), energy-dispersive X-ray (EDX) spectrometry, thermogravimetric analysis (TGA), X-ray diffraction (WAXD) and Fourier-transform infrared (FTIR) spectroscopy. The optimization of an original procedure to reduce the dimensions of the ashes in an aqueous solution was then developed by using ball milling and sonication techniques, and the nanometric dimensions of the particles dispersed in water were estimated by dynamic light scattering (DLS) measurements in the order of 300 nm. Finally, possible industrial applications for the nanomaterials obtained from the waste ashes are suggested, including, for example, inks for Aerosol Jet^®^ Printing (AJ^®^ P).

## 1. Introduction

During the last decade, the circular economy has become an important domain of academic research, with a sharp increase in the number of articles and journals covering this topic [1]. Geissdoerfer et al. [1], as well as Schut et al. [2], claim that the most significant circular economy definition has been provided by Ellen MacArthur Foundation [3,4]. The use of wastes, including wastes from industrial processes, such as carbon-based ashes (CA), is, therefore, an important development objective of the circular economy [5].

The management of carbon waste ashes still represents an issue. Even though significant amounts of CA are used in various applications, for example, as a substitute for cement in concrete, [6,7], large quantities of them are not utilized and are disposed of in landfills [6], with long-term negative effects on the environment and human health. CA indeed contribute to the formation of particulate matter (PM), which is one of the major causes of airborne pollution and has been found to induce different cancers, cardiovascular diseases and reproductive disorders [8]. Hence, it would become necessary to regard CA as raw material that can be converted into new products rather than waste.

In the last few years, several studies have proposed the reuse of carbon waste ashes to obtain nanomaterials for different applications. As an example, Ramanathan et al. investigated the use of CA, obtained as a byproduct of the coal combustion from power plants, for the synthesis of nanosized particles due to their enrichment in silica, kaolin, iron, and alumina. In this regard, the nanocrystalline aluminosilicates are one of the most promising nanocomposites synthesized starting from CA through the alkaline treatment method. Their main applications are in fields such as wastewater treatment, agriculture system, and antioxidants, although their use is being evaluated in the medical field applications, especially in drug delivery and delivery systems, bone engineering, biosensors, hemodialysis and intestinal therapies [9]. In addition, Salah et al. investigated the production of carbon nanotubes (CNTs) using ultrasonicated CA, obtained by the heavy oil combustion from water desalination plants, as precursors and catalysts [10,11]. These authors also reported on the preparation of carbon nanoparticles (CNPs) using the ball milling technique [12]. These CNPs were useful for various applications, including fillers in epoxy composites, additives in lubricant oil, gas adsorption, etc. [12,13]. Schlatter et al. also investigated the use of carbon black, derived from the combustion of heavy petroleum products, in inkjet printing for the production of electronic devices [14]. However, to the best author’s knowledge, no evidence has been reported so far regarding the reuse of CA, derived by power plants for domestic and public use, and/or its use for the development of 3D printing materials.

In this study, an original and green procedure to produce water-based solutions containing nanometric recycled carbon particles is proposed. CA, used in this work, is produced by the wooden biomass pyro-gasification plant CMD ECO20. These plants and processes are applied in the production of electric power and heat for domestic and small public facilities. Overall CA production can be estimated in ~1 kg/h (5 wt % of biomass introduced into the plant) [15]. Similar to coal ashes reported in [9,10,11,12,13], biomass ashes are a multi-component system of powder material [16]. Nevertheless, they show contents of Ag, Au, B, Be, Cd, Cr, Cu, Mn, Ni, Rb, Se and Zn, which are higher than the respective Clarke values (worldwide average contents) for coal ashes [16], making them particularly attractive for applications, such as, production of construction materials and sorbents, but also synthesis and production of minerals, ceramics and others. Hence, the CMD CA were, first, characterized by means of several techniques, including microcomputed tomography (microCT), differential scanning calorimetry (DSC), scanning electron microscopy (SEM), energy-dispersive X-ray (EDX) spectrometry, thermogravimetric analysis (TGA), wide-angle X-ray diffraction (WAXD) and Fourier-transform infrared (FTIR) spectroscopy, in order to identify their composition, size, morphology and material properties. The ashes are then treated by various techniques in order to reach a nanometric size, as requested by the typical applications. The dispersion obtained is finally characterized by dynamic light scattering (DLS). In conclusion, a possible application for the obtained carbon-based water solution as an ink component for Aerosol Jet^®^ Printing (AJ^®^P) is proposed. AJ^®^P is a nozzle-based additive manufacturing technology of the direct writing family, aiming at the production of fine features on a wide range of substrates. Originally developed for the manufacture of electronic circuits, AJ^®^P has been investigated for a range of applications, including active and passive electronic components, actuators, sensors, as well as a variety of selective chemical and biological responses [17]. The use of water-based solutions containing carbon nanomaterials, such as carbon nanotubes [18], [19,20], graphene oxide [21] and graphene [22], for AJ^®^P is known in the literature. However, to the best of the author’s knowledge, recycled carbon nanoparticles aqueous inks have never been using before in AJ^®^P.

## 2. Materials and Methods

### 2.1. Generation of the Byproduct (CA)

Carbon-based ashes (CA) are the byproduct of the biomass pyro-gasification plant CMD ECO20, developed by the company Costruzioni Motori Diesel S.p.A. (CMD) to produce electric power and heat, starting from woodchips waste. The CMD ECO20 plant is an integrated system combining a downdraft gasifier, a spark-ignition internal combustion engine (ICE), an electric generator and syngas cleaning devices. This system processes wooden biomass of G30 size (1.50 to 3.00 cm) and max at 20% of humidity. It can produce electricity and heat up to 20 kWe and 40 kWth, and it is a computerized machine managed at every level of operation [23] and used in domestic and consumer applications. CA are collected and recovered from the dust collector downstream of the plant.

### 2.2. Characterization of the Byproduct (CA)

The properties of the CA were characterized under various aspects. The ashes collected from the downstream of the plant will be referred to as neat CA. The thermal conductivity of the neat CA was calculated by means of a differential scanning calorimetry (DSC) (Mettler Toledo, Columbus, OH, USA) by using a sensor material (indium), whose melting temperature was 156.6 °C. A neat CA sample was placed into an aluminum crucible, and the indium sensor was put on up the sample. A single scan from 25 °C to 250 °C at a heating rate of 10 °C/min in a nitrogen atmosphere was performed on at least three samples. By considering the method of Flynn and Levin [24], the slopes of the indium and sample endothermic peaks were calculated in order to determine the resistance of the sample (Rs) as follows:

(1) $$\mathrm{Rs}=\;R^{\prime }-R$$

where R is the thermal resistance between calorimeter and indium, R′ is the thermal resistance between calorimeter and indium with the sample. The thermal conductivity (k) is determined by Equation (2):

(2) $$k=\;\frac{L}{A\;\left(\;R^{\prime }-R\right)}=\frac{L}{{A\;R}_{s}}$$

where L is the sample height, A is the contact area between sample and sensor material.

The porosity of the neat CA was estimated by means of a pycnometer according to Equation (3):

(3) $$\rho =\frac{m}{V}$$

where the m is the mass of the sample and V is the volume calculated according to Equation (4):

(4) $$V=\frac{\left(m_{w}+m_{s}\right)-m_{\mathrm{ws}}}{\rho_{w}}$$

where m_w_ is the mass of the water inside the pycnometer, m_s_ is the dried weight of the sample, m_ws_ is the mass of the system when the sample is immersed in the water, and ρ_w_ is the density of water (1 g/cm^3^).

The microcomputed tomography (microCT) was performed to evaluate the porosity of the neat CA by using a Bruker SkyScan 1172 (Bruker Corporation, Billerica, MA, USA). The following microCT settings were identified for proper scanning of samples, based on their X-ray attenuation capacity: (a) the X-ray source was powered at 50 kV and 200 μA; (b) a 0.5 mm Al filter was used; (c) for the detection of porosity, the pixel size was set at 10 μm; (d) the exposure time was 600 ms, with a 2 × 2 binning; (e) samples were rotated 360°, with a rotation step of 0.4°. After reconstruction with NRecon software, DataViewer was used to visualize the 3D sections of the samples in the XY, XZ and YZ planes. The reconstructed grayscale images of the samples were then analyzed with CTAn software to quantify the porosity after selection of a volume of interest (a cylindrical Volume of Interest-VOI with 5.3 mm diameter and 1 mm height) and appropriate thresholding.

In order to further characterize the neat carbon-based ashes, they were initially reduced in rough powder by using a milling mortar. The ashes treated by milling mortar will be referred to as mortar-milled CA.

The morphology of the mortar milled CA was investigated by scanning microscopy (SEM) using a Zeiss scanning electron microscope Evo40 (Carl Zeiss Microscopy, LLC, White Plains, NY, USA). The elemental analysis of the particles was performed by energy-dispersive X-ray (EDX) spectroscopy using a Bruker, XFlash detector 5010 (Bruker Corporation, Billerica, MA, USA).

Thermogravimetric analysis (TGA) of the mortar milled CA was carried out using a TGA TA instrument SDT Q600 (TA Instruments, New Castle, DE, USA).to confirm the elemental composition. About 10 mg of powder samples were heated in an alumina holder under a nitrogen atmosphere from 20 to 900 °C at a heating rate of 10 °C/min.

The structure of the mortar milled CA was obtained by wide-angle X-ray diffraction (WAXD) using an automatic Bruker D2 Phaser diffractometer (Bruker Corporation, Billerica, MA, USA), in reflection mode, at 35 KV and 40 mA, using nickel-filtered Cu-K radiation (1.5418 Å).

The chemical nature of the functional groups was studied by Fourier-transform infrared (FTIR) spectroscopy of the mortar milled CA using a BRUKER Vertex70 spectrometer (Bruker Corporation, Billerica, MA, USA) equipped with a deuterated triglycine sulfate (DTGS) detector and a KBr beam splitter, at a resolution of 2.0 cm^−1^. The frequency scale was calibrated to 0.01 cm^−1^ using a He–Ne laser. In total, 32 scans were signal averaged to reduce the noise, and the spectrum of the ashes was collected using KBr pellets.

### 2.3. Preparation and Characterization of the Aqueous Solution Containing CA-Based Nanoproducts

After the characterization of the CA as raw material (both before and after the mortar milling treatment), the work aimed at reducing the carbon-based ashes to nanometer size in an aqueous solution and investigating their potential for target applications. For this purpose, multistep reduction size processes were developed, monitoring the size and the distribution of the treated CA ashes by multi-angle laser scattering (MALS) (CILAS 1190 particles size analyzer) (CPS Us, Inc., Madison, WI, USA) and by dynamic light scattering (DLS Zetasizer-Malvern) (Malvern Panalytical Ltd, Malvern, UK). In this work, the reduction processes are divided into two principal methods, labeled hereafter, as method I and method II. The ball milling was carried out in an aluminous porcelain jar (1.5 L), using alumina balls in an ambient atmosphere; the mechanical milling was performed in a horizontal oscillatory mill MMS-Ball Mill (M.M.S.2 S.r.l, Nonantola (Modena), Italy), operating at 40 Hz.

The first method, method I, has involved different steps. First of all, the mortar milled CA ashes were dispersed in water. Second, this water-based suspension was ball milled and centrifugated. Finally, the supernatant of the treated CA/water dispersion was sonicated. Based on the results of method I, a second procedure, method II, was proposed in order to reduce the time of the process, improve the efficiency of the size reduction and increase the yield of the process. Therefore, according to method II, the mortar-milled CA were first, dry ball-milled for 24 h and successively dispersed in water and ball-milled for a strongly reduced time (498 h and 24 h, for method I and method II, respectively). After this, a sonication step of the water-CA dispersion was added before the centrifugation step. The time of each step of both methods was established after several experimental measurements of the CA size, as well as the time necessary to obtain the maximum size reduction of the ashes by using the methodology proposed for each step. In detail, the duration of each processing step was identified based on the minimum time necessary to obtain the maximum reduction in size, where the minimum time is identified when two consecutive measurements gave constant size values. In method II, similar micrometric values have been reached in advance compared to method I, significantly reducing the time of treatment. A schematic list of each step of both methods is reported as follows: According to method I, after mortar milling, the CA were dispersed in water and reduced by means of the following protocol:

- Wet ball milling for 498 h (CA in water: 50 g/L);
- Centrifuge at 10,000 rpm for 20 min;
- Sonication of the supernatant for 5 h (by ultrasonic bath CP104) (CA in water: 3 g/L).

According to method II, after mortar milling, the CA were treated by means of the following protocol:

- Dry ball milling for 24 h;
- Wet ball milling for 24 h (CA in water: 50 g/L);
- Sonication for 32 h (by Diagenode Bioruptor Plus sonication device);
- Centrifuge at 10,000 rpm for 20 min;
- Sonication of the supernatant for 5 h (by ultrasonic bath CP104) (CA in water: 4 g/L).

## 3. Results

**Figure 1:**
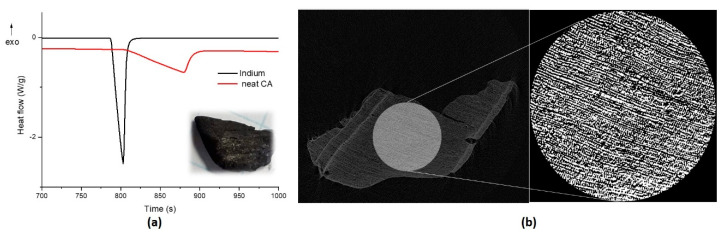
(**a**) Calorimetric curves of thermal conductivity measurements and (**b**) microCT images of neat carbon-based ashes (CA) sample.

The neat CA collected by the CMD plant (inset of Figure 1a) are rough and porous particles of irregular shape. The thermal conductivity was measured by DSC analysis (Figure 1a), and it was equal to 0.292 ± 0.003 W/mK. Those values are in line with the ones reported in the literature [25] related to the thermal conductivity of carbon black (0.2–0.3 W/mK), which is widely used as a thermal insulator. From pycnometer measurements, the density was estimated as 0.3 g/cm^3^ via Equation (3), and the rough surface was also evinced by microCT analyses (Figure 1b); finally, the quantitative microCT 3D analysis (Figure 1b) revealed an overall porosity of 51.17%.

**Figure 2:**
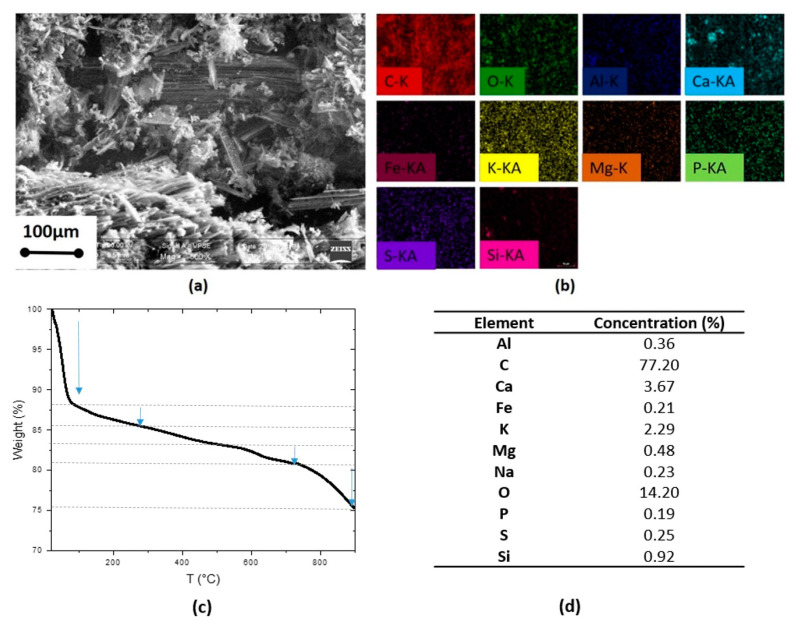
(**a**) SEM image, (**b**) EDX mapping, (**c**) TGA plot whit arrows indicating steps of weight losses and (**d**) EDX data of the carbon-based ashes after mortar milling (mortar milled CA).

Figure 2a–d reports the results on morphological inspection and composition analysis of the milled carbon-based ashes (CA) obtained as a waste product from pyro-gasification of woodchip after initial mortar milling treatment.

The morphological data acquired by scanning electron microscopy (SEM) (Figure 2a) shows particles/fragments with an elongated shape and a minimum average long size of about 80 μm. It is to note that this type of ashes is larger in size than the mortar milled ashes, obtained from previous research conducted by the authors on a different innovative green waste reported in [23], also generated by the CMD plant.

The elements analysis performed through the energy-dispersive X-ray (EDX) equipment (Figure 2b) shows that the most abundant element of the CA is carbon, which is present in a percentage of about 77%. Several other atomic elements can also be observed at different concentrations. The most plentiful elements (>1%), besides carbon, are O, Ca and K. Small amounts (<1%) of Al, Fe, Mg, Na, P, S and Si are also present on the ashes surface. The elements characterization evidenced a composition of the ashes from pyro-gasification of woodchips remarkably close to the ashes from pyro-gasification of innovative green waste used in [26] despite the different initial waste products.

The variety of the mortar milled CA composition was further confirmed by thermogravimetric analyses. Indeed, the TGA curve reported in Figure 2c entails several weight loss steps due to the variety of the neat CA composition. In particular, the first step of about 12%, ranging from room temperature to about 100 °C, is attributed to the removal of the molecularly adsorbed water. A second step (about 3%) between 100 °C and 200 °C, is originated from the removal of the thermally labile oxygen-containing functional groups. Another step of about 2% ranging from 580 to about 700 °C, can be due to the decomposition of CaCO_3_ [27]. A residual weight of about 75% is observed at 900 °C showing that the main component of the ashes is carbon, as also confirmed by the EDX data.

**Figure 3:**
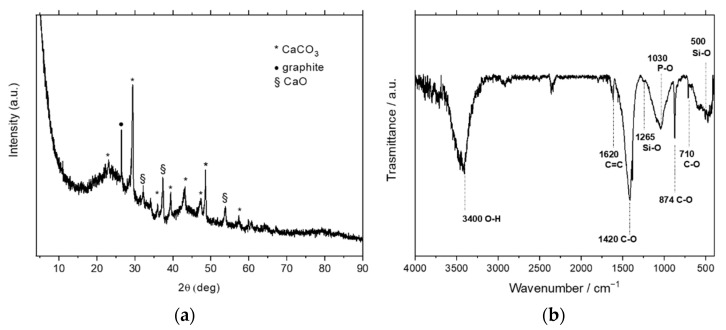
(**a**) X-ray diffraction and (**b**) FTIR spectroscopy of the mortar milled CA ashes.

The structural and spectroscopic analysis of mortar milled ashes were made by wide-angle X-ray diffraction (WAXD) and Fourier-transform infrared (FTIR) spectroscopy, as reported in Figure 3a,b, respectively.

The spectra in Figure 3a show several crystalline peaks, which can be attributed to calcium carbonate (CaCO_3_) and calcium oxide (CaO), and the typical peak of graphite at 2θ = 26.5° [28]. The diffractometric results are confirmed by FTIR spectra of Figure 3b, where the principal bands related to calcite (1420, 874, 710 cm^−1^), but also magnesium carbonate (1440, 1375 cm^−1^), silicates (1260–800 cm^−1^ stretching and 500 cm^−1^ bending) and phosphates (around 1030 cm^−1^), detected in traces by WAXD, were identified by spectroscopic analysis as reported in [29] for biomass ashes. Moreover, the peak at 3400 cm^−1^ associated with the O-H stretching of the absorbed water confirms the TGA results.

**Figure 4:**
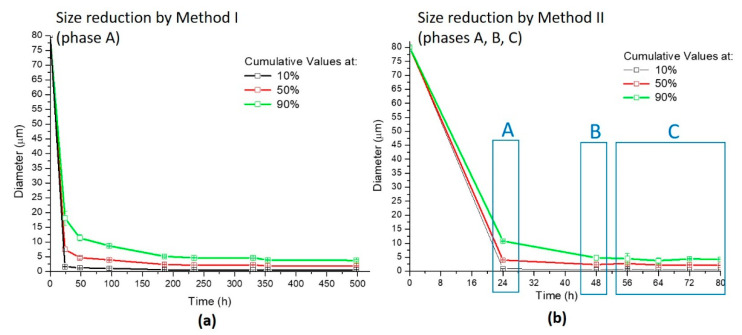
(**a**) Multi-angle laser scattering (MALS) data of the reduced CA during phase A of method I; (**b**) MALS data of the reduced CA during phases A, B and C of method II.

The reduction of particle size of mortar-milled CA for both methods, method I and method II, was evaluated in each phase at regular intervals of time. The results obtained in the first phases of the methods (phase A for method I and phases A, B, C for method II) are reported in Figure 4.

As evidenced by the graphs in Figure 4, the granulometric size of the CA was different for methods I and II. In fact, just after 24 h of dry ball milling (phase A of method II—Figure 4b), the cumulative values at 10%, 50% and 90% of the distributions were lower than the values recorded after 48 h of wet ball milling of method I (Figure 4a). After 48 h (24 h dry ball milling +24 h wet ball milling, i.e., phase A+B in method II), the treated CA particles reached values close to those treated after 186 h by wet ball milling in method I. The same trend was visible throughout the entire curve. In addition, the size of the reduced particles obtained via the steps of Figure 4 are comparable, being the mean diameters equal to (2.03 ± 0.17) µm in method I and (2.28 ± 0.08) µm in method II. Nevertheless, method II presents a great advantage in terms of efficiency, reaching comparable resulted to method I in a fraction of time (about 1/6). At this stage, however, none of the two procedures was able to reach a satisfactory quantity of nanometric particles, as from the typical request of industrial applications based on carbon powders. Hence, an additional reducing step was introduced, and the CA treated by methods I and II of Figure 4 were centrifuged at 1000 rpm for 20 min (phase B of method I and phase D of method II, Section 2.3). The results of the granulometric analysis of the supernatants taken after centrifuge are reported in Table 1.

**Figure 5:**
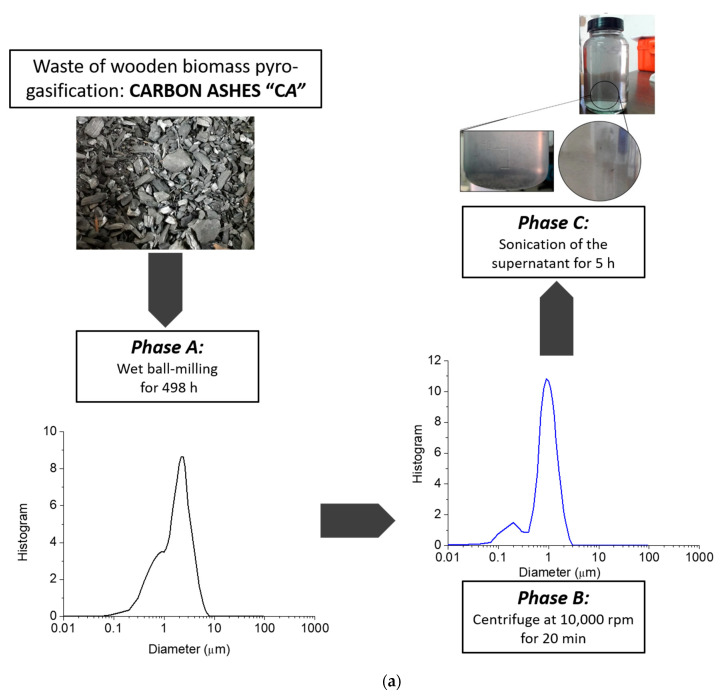

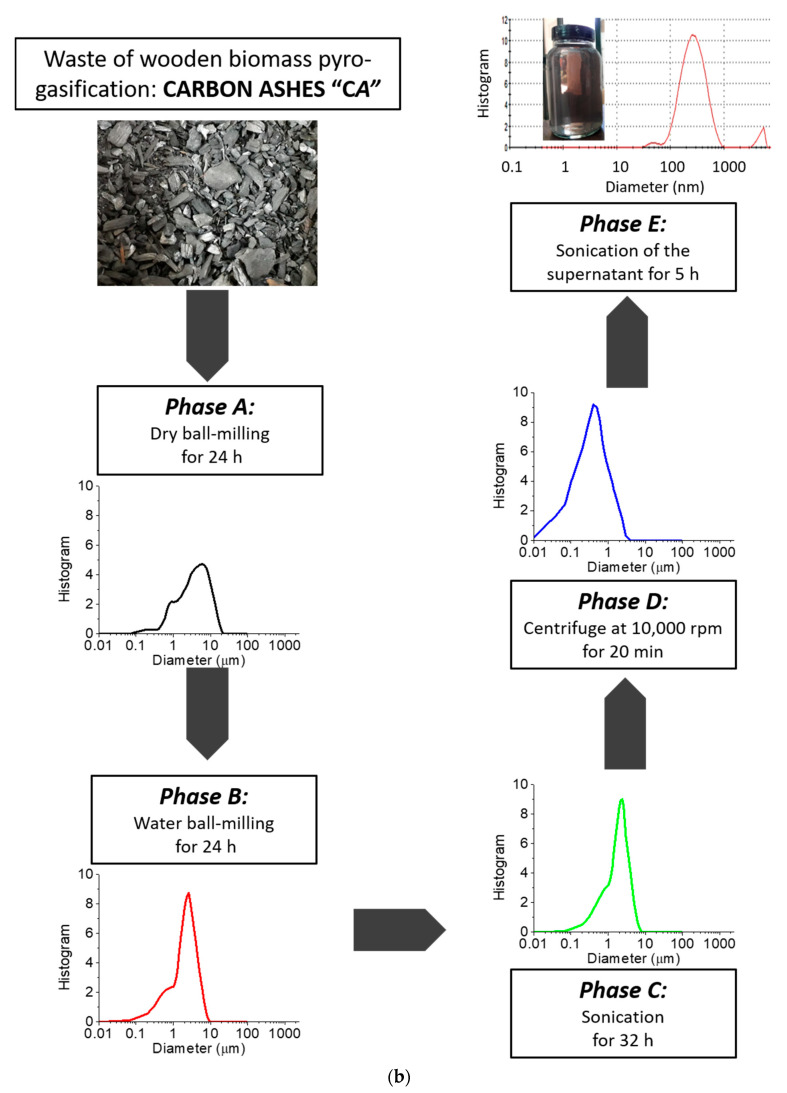
Diagrams of the (**a**) rejected method I and of the (**b**) method II, developed as best practice for the nanometric reduction of carbon ashes generated by pyro-gasification of woodchips and corresponding granulometric analysis.

Despite the very small size achieved, the majority of the particles were still of micrometer size (90% cumulative value, Table 1). The supernatants were then again treated and subjected to sonication for 5 h in an ultrasonic bath (phases C and E of method I and method II, respectively). It was observed that sonication caused aggregation of the CA particles obtained from method I, changing the esthetical aspect of the solution also at the naked eye (Figure 5a). Instead, the dispersion obtained with treated CA derived from method II appeared stable and homogeneous, revealing particles with a diameter of (331.3 ± 188.7) nm at 98.4% of the distribution, as recorded by means of DLS analysis (Figure 5b). The method II procedure was then revealed to be a simple, ecological, and efficient method able to reduce the carbon ashes from pyro-gasification of wooden biomass to nanometer size particles, dispersed in water in an average concentration of 0.4 wt %. Starting from these preliminary results, method II was selected as the most efficient procedure to obtain aqueous dispersions based on nanometric CA ashes. A representation of the identified optimal procedure (method II) of this paper, step-by-step, along with the granulometric distribution of each phase, is reported in Figure 5b.

**Figure 6:**
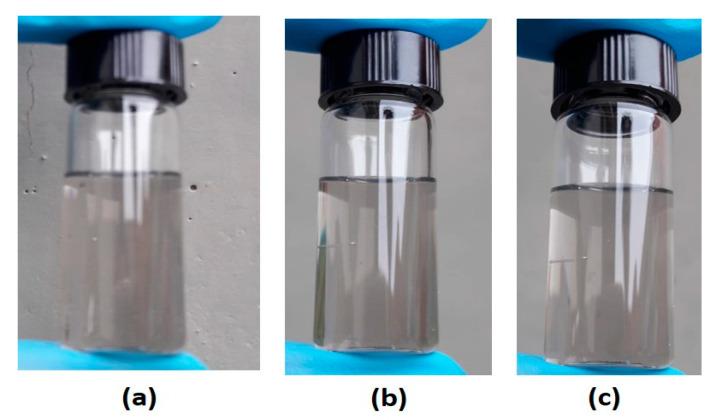
The appearance of Costruzioni Motori Diesel S.p.A. (CMD)-nanoCA-based water dispersion, obtained by method II, after (**a**) 0, (**b**) 30 and (**c**) 90 days.

Method II, developed in this paper, demonstrated to be able not only to obtain several liters of the water-based dispersions but also to produce dispersions with a reproducible nanometric size. In addition, the nano-based dispersion, obtained by using method II, was also revealed to be stable over time, as demonstrated by the images in Figure 6.

**Figure 7:**
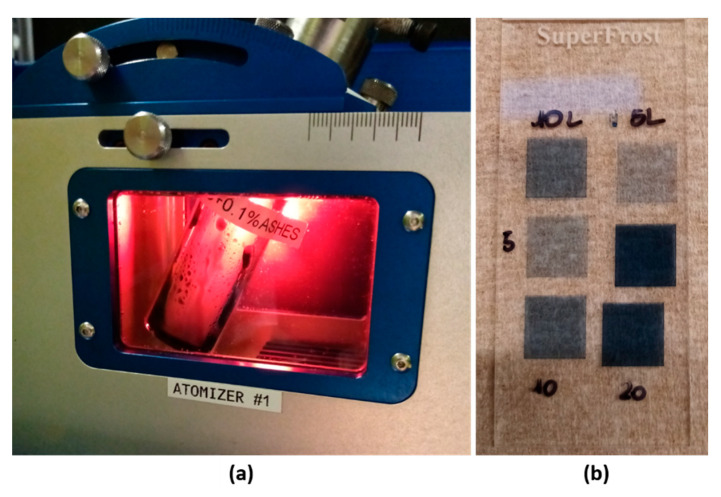
(**a**) Ultrasonic atomization in AJ^®^P setup and (**b**) pattern samples printed onto glass slides of poly(3,4-ethylenedioxythiophene)-poly(styrenesulfonate) (PEDOT:PSS) and nano-CA water-based dispersion with nano-CA concentrations of 0.1% wt/V_PEDOT:PSS_.

As a provisional study, two inks containing a commercial poly(3,4-ethylenedioxythiophene)-poly(styrenesulfonate) (PEDOT:PSS) water-based ink (Orgacon^®^ IJ 1005, Agfa, Mortsel, Antwerpen, Belgium and nano-CA water-based dispersion, obtained by the selected method II, were prepared with nano-CA concentrations of 0.05% and 0.1% wt/V_PEDOT:PSS_, respectively. The two inks (each one of ~1 mL) were successfully ultrasonically atomized (Figure 7a) in an AJ^®^P setup (Optomec, 300s system).

## 4. Discussion

The neat carbon-based ashes (CA) used in this study are produced by the CMD ECO20 plant. This system uses wooden biomass as fuel to provide energy efficiency and environmental benefits by reducing the consumption of fossil fuels and associated greenhouse gas emissions [15]. It finds application in the production of heat and electric power for domestic and public use. The byproduct is a porous, rough carbon ash particle of irregular shape (Figure 1), which was found to have low thermal conductivity (<0.3 W/mK), similar to one of the carbons black, despite the carbon nature. The result is most likely due to the high porosity of the particle collected by the plant. In particular, the density and overall porosity of the neat CA particles of this work were here estimated by pycnometer measurements and microCT analysis, like 0.3 g/cm^3^ and 51.17%, respectively. As reported by Wall et al. [30], the thermal conductivity of ash deposits depends in fact not only on the chemical composition, where magnesium or siliceous oxides decrease or increase the thermal conductivity, respectively, but also on the physical state and texture, and the temperature of the deposit. The high porosity of the ashes could be ascribed to the down to few mbar pressures reached during the sintering process. The lack of suitable conditions can, in fact, promote a possible gas production causing the formation of a highly porous structure, as come about to intercalated graphite after the expansion process. Some of the authors studied in previous work [31] how the fast heating at 700 °C for 2 min of the intercalated graphite promoted the formation of a worm-like shape and highly porous microstructure due to the sulfuric acid decomposition and to the formation of intercalated galleries.

The neat CA particles were then pretreated via mortar milling and characterized under various aspects. In particular, the mortar milled ashes composition was found to be very similar to the ones obtained by the CMD plant with different biomass in input, while the size was found to be larger since although both have a lignocellulosic nature, they differ in shape and size, and it is known in the literature that the biomass combustion ashes keep the same morphology of the starting biomass [16], affecting the result obtained by hand milling.

The mortar-milled CA were then further treated to nanometer size, and an original and green procedure to reduce their dimensions was developed (method II) by using various techniques, such as ball milling and sonication, and water-based solutions. Method II was selected among the two procedures explored, as it presents several advantages. The most important advantage is the possibility to obtain stable and reproducible water dispersed nanometric CA-based inks in a shorter time, compared to the time required by method I. This is possible thanks to the initial dry phase, in which the particle size is drastically reduced already within the first 24 h. A further advantage is the possibility to produce carbon-based nanoparticles dispersed in a green solvent (water). In fact, these can be used for many potential industrial applications, or at least for easier, more economic, and more ecological disposal of the nanometric ashes, compared to the neat wastes. Method II was, hence used to produce large amounts (about 5 L) of nanometric water-based dispersions that will be investigated for different potential applications. However, the yield of the method II process is still too low, and this aspect requires further studies. A possible improvement of the proposed methodology could be obtained by using a cryomilling technique by milling the starting mortar milled powders within LN2 with milling balls forming a slurry during milling. This process has been successfully applied in the literature to reduce the dimensions of thermoplastic polymers, and it is known as “cryogenic attrition” [32]. This approach will be tested in a further paper.

Although a low concentration of carbon nanoparticles in aqueous solution (0.4 wt %) could be obtained, by the selected optimal method, method II, these innovative water-based dispersions of nanometric CA can attract considerable attention as material candidates for potential applications in additive manufacturing (AM) or coating technologies and in the sequestration of carbon dioxide by aqueous carbonation [33]. The main features of the proposed CA refer to (i) a particle size around 300 nm for a total of ~98%, (ii) a high presence of carbon (~77%), and (iii) a thermal conductivity like carbon black.

In the context of AM, nanoscale CA dispersions may have a competitive advantage as water-based fillers for the formulation of inks in different printing techniques, such as direct writing (DW) inkjet printing (viscosity range (10–20) mPas, particle size limited by the nozzle diameter, usually ≤200 nm), AJ^®^P (viscosity range (1–1000) mPas, particle size ≤0.5 μm) and syringe extrusion-based (viscosity range usually (100–10^4^) Pas, particle size-dependent by the nozzle, usually ≤100 μm) printing [34].

Inks and film coatings, such as organic polymers or ceramics, are indeed widely used in printed electronics (PE) and in the semiconductor industry for conductive circuits (e.g., sensors, thin-film transistors, etc.). Moreover, the embedment of CA nanoparticles into hydrogel-based compositions, such as soft biomaterials or polymers, may increase the mechanical rigidity of the overall structure over time. This property may be useful in biomedical and tissue engineering applications, such as scaffold development for in vitro or in vivo studies.

In this study, two inks, composed of a poly(3,4-ethylenedioxythiophene)-poly(styrenesulfonate) (PEDOT:PSS) water-based ink (Orgacon^®^ IJ 1005, Agfa, BE) and nanometric CA water-based dispersion with nano-CA concentrations of 0.05% and 0.1% wt/V_PEDOT:PSS_ were, finally, prepared, in order to verify the potential applications of the nanometric waste ashes. The two CA-based inks were, even, successfully ultrasonically atomized by using an AJ^®^P setup (Figure 7a). In both cases, homogenous and stable atomization was achieved at power atomization, P = 45 V. Subsequently, pattern samples (squared shape 8 × 8 mm, with serpentine filling) (Figure 7b) were printed onto glass slides (Superfrost^®,^ VWR, Leuven, Belgium at platen temperature 60 °C, printing speed 15 mm/s, carrier and sheath gases flow 30 sccm, and a number of layers equal to 5, 10 and 20. No visual differences in the mist generated were detected over the printing period, demonstrating the stability of the inks. A post-sintering process was implemented in a thermal oven (Heraeus, Hanau, Germany at 150 °C for 45 min. Evaluation of sample properties in terms of print quality, thermal and electrical conductivity is currently ongoing. Therefore, future studies will be towards a more comprehensive evaluation of CA performance towards the target application.

In both cases, excellent adhesion on the desired substrate, high-frequency stability, high permittivity, and good biocompatibility (for biomedical purposes) is required.

In addition, a green application, such as the sequestration of CO_2_ in aqueous carbonation, will be investigated. The increasing CO_2_ concentration in the Earth’s atmosphere is indeed one of the main causes of global warming. Therefore, a technology that could contribute to reducing carbon dioxide emissions is the ex situ mineral sequestration (controlled industrial reactors) of CO_2_ [33].

## 5. Conclusions

In this manuscript, carbon-based ashes (CA) obtained as a waste product from pyro-gasification of woodchip were characterized by several techniques and treated to reduction to nanometer size for exploitation in industrial applications. The thermal conductivity measurements of the neat CA showed values similar to carbon black, widely used as a thermal insulator, while the microCT analysis highlighted an overall porosity of 51.17%. The scanning electron microscopy (SEM) of the mortar milled CA showed particles/fragments with an elongated shape and a minimum average long size of about 80 μm. The energy-dispersive X-ray (EDX) spectroscopy also demonstrated that the most abundant element of mortar milled CA is carbon, present in a percentage of about 77%, and the most plentiful elements (>1%), besides carbon, are O, Ca and K. Moreover, the thermogravimetric analysis (TGA) highlighted the presence of several weight losses steps due to the variety of carbon ashes composition: in particular, a residual weight of about 75% at 900 °C, ascribed to the main component of the ashes, which is carbon, as also confirmed by EDX data. Finally, the wide-angle X-ray diffraction (WAXD) revealed several crystalline peaks attributed to calcium carbonate and calcium oxide and the typical peak of graphite. These diffractometric results were then confirmed by FTIR spectra.

Afterward, nanometric CA water-based dispersions were obtained by means of an innovative multistep reduction size process in order to propose a potential industrial application for the nanomaterials, obtained from carbon waste ashes, as inks for additive manufacturing (AM) techniques, such as nozzle-based direct writing (DW) AJ^®^P.

## Figures and Tables

**Table 1:** Granulometric data of the supernatants were obtained from both methods I and II after centrifuge.

Samples	Cumulative Value at 10% of the Granulometric Distribution (µm)	Cumulative Value at 50% of the Granulometric Distribution (µm)	Cumulative Value at 90% of the Granulometric Distribution (µm)	Mean Diameter (µm)
**CA reduced by method I**	0.20 ± 0.05	0.96 ± 0.04	1.79 ± 0.09	1.00 ± 0.03
**CA reduced by method II**	0.10 ± 0.02	0.61 ± 0.28	1.48 ± 0.23	0.69 ± 0.16
